# Giant Cell Arteritis With Mesenteric Involvement and Superimposed Functional Gut Syndrome: A Case Report

**DOI:** 10.1002/ccr3.72643

**Published:** 2026-05-04

**Authors:** Benjamin Rao, Ronan O'Connor

**Affiliations:** ^1^ Department of Medicine Deakin University Geelong Victoria Australia; ^2^ Department of General Medicine Eastern Health Melbourne Victoria Australia; ^3^ Department of General Medicine Monash Health Clayton Victoria Australia; ^4^ Department of Gastroenterology Eastern Health Melbourne Victoria Australia

**Keywords:** extracranial giant cell arteritis, functional gut syndrome, giant cell arteritis, mesenteric ischemia

## Abstract

Giant cell arteritis‐associated mesenteric vasculitis is a rare manifestation and often carries significant mortality and morbidity. We present a case of GCA with mesenteric involvement that was at first diagnostically challenging, contributed to by a history of functional illness; however, it achieved successful treatment without ischemic complications.

## Graphical abstract

FDG PET‐CT images of giant cell arteritis with mesenteric involvement.

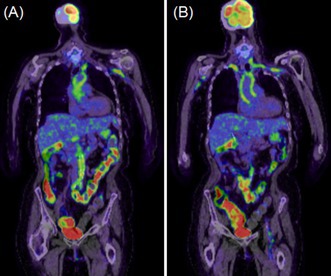

## 1 Introduction

Giant cell arteritis (GCA) is a systemic vasculitis of large and medium size vessels [1]. Presentation depends on the vessels involved; however, typical constitutional symptoms include fatigue, weight loss, and fever. Classical temporal arteritis manifestations include jaw claudication and temporal headache, while more concerning features for cranial ischaemia include vision loss and stroke symptoms [2]. Isolated extracranial or nonspecific manifestations without cranial involvement are present in a significant minority of cases, and can present as limb claudication, isolated fever, or other large vessel involvement, making diagnosis challenging [3].

Investigations include raised inflammatory markers, while diagnosis is supported by mural thickening and giant cells on temporal artery biopsy (TAB), temporal artery ultrasound assessment with doppler, or positron emission tomography (PET) scan for extracranial disease [4].

GCA with involvement of the mesenteric arteries is uncommon and is associated with significant morbidity and mortality [5]. Here we present a case of GCA with mesenteric involvement.

## 2 Case History and Examination

A 69‐year‐old female was admitted to general medicine with a 6‐month history of progressive postprandial abdominal pain and weight loss. The patient had a past history of functional gut disorder and irritable bowel syndrome of mixed phenotype known to a multidisciplinary clinic, with a previous unremarkable colonoscopy, uncontrolled migraines, and prior appendectomy and cholecystectomy.

The history was significant for subacute weight loss of 8 kg alongside intermittent night sweats, without fevers, myalgia, or arthralgia. The patient reported ongoing variable bowel habits without blood or mucus, and she had a persistent frontal headache similar to previous episodes, without visual disturbance.

On review, the patient was afebrile with no appreciable lymphadenopathy, the abdomen was soft and diffusely tender, and there was no temporal artery tenderness with pulses appreciable bilaterally.

## 3 Investigations

Laboratory testing revealed a C‐reactive protein (CRP) of 250 mg/L, erythrocyte sedimentation rate of 120 mm/h with mild neutrophilia of 8.7 10^9^/L. The patient had anemia of chronic disease with hemoglobin and ferritin values of 85 g/L and 352 µg/L, respectively. An autoimmune screen was negative for antinuclear antibody, extractable nuclear antibodies (ENA), anti‐double stranded DNA (dsDNA), rheumatoid factor, and anti‐neutrophil cytoplasmic antibodies. Blood, urine, and stool cultures showed no significant growth at 5 days.

Computerized tomography (CT) of the abdomen and pelvis post‐contrast suggested mild left‐sided colitis with no abnormal wall thickening and patent splanchnic vessels.

The patient received a PET‐CT with fluorodeoxyglucose, which revealed large vessel vasculitis involving the thoracic and abdominal aorta, subclavian and axillary arteries, superior mesenteric artery (SMA), common carotids, and common iliac arteries. There was diffuse avidity in the colon from caecum to mid‐descending colon (Figure 1).

**FIGURE 1:**
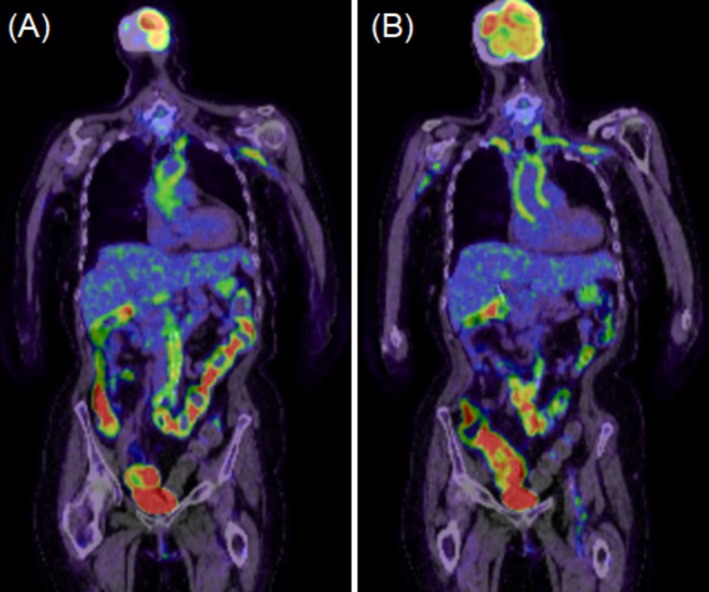
FDG PET‐CT showing increased uptake in the large colon (A), and large vessels including the subclavian and axillary arteries (B).

## 4 Diagnosis, Outcome and Follow‐Up

A diagnosis of extracranial giant cell arteritis with mesenteric involvement was confirmed from radiological and biochemical evidence, and daily prednisolone 1 mg/kg oral was commenced. Bilateral temporal artery biopsies were performed on Day 7 post prednisolone commencement, but revealed no evidence of inflammation or giant cells on histology.

Further investigation for colitis included a raised fecal calprotectin of 696 µg/g. Flexible sigmoidoscopy, converted to a full colonoscopy opportunistically on an unprepared colon, showed no abnormalities, and biopsies were negative for inflammation. Outpatient gastroscopy revealed chronic gastritis, while repeat colonoscopy showed patchy melanosis coli and no inflammation; however, poor bowel preparation limited assessment.

On discharge, CRP was below 10 mg/L, with a weaning course of prednisolone to reduce by 5–10 mg every week. At 2‐week follow‐up and 1 month post‐steroid commencement, the patient reported no improvement in symptoms, and tocilizumab was commenced to facilitate steroid wean. At 1‐month and 2‐month follow‐up, the patient achieved good symptomatic improvement.

## 5 Discussion

While GCA is considered the most common form of vasculitis affecting medium and large vessels, incidence varies significantly by region, with rates of 10–40 per 100,000 people in Northern Europe [1]. GCA is uncommon under the age of 50, with an increasing incidence into the eighth decade of life [6]. Scandinavian descent and white background are major risk factors, and the condition is more common in females, with an approximate 2–3:1 incidence compared to males [2].

Extracranial GCA, also known as large‐vessel GCA (LV‐GCA), is a subtype of GCA involving inflammation of the aorta and its major proximal branches (e.g., subclavian, axillary, carotid, vertebral arteries), with or without concurrent cranial involvement. Unlike classic cranial GCA, which features prominent head and neck symptoms, extracranial forms often present with nonspecific systemic symptoms such as fever, weight loss, fatigue, night sweats, polymyalgia rheumatica‐like shoulder/hip girdle pain and stiffness, or limb claudication (e.g., arm fatigue or ischemia from upper extremity vessel stenosis) [7]. Purely extracranial (isolated LV‐GCA) cases lack typical cranial signs like headache, jaw claudication, scalp tenderness, or visual loss, leading to diagnostic delays [8].

The diagnosis of GCA relies on supportive imaging, including ultrasound and PET‐CT, and tissue biopsy, typically from bilateral temporal arteries [4]. TAB positivity rates are often lower in LV‐GCA patients [3, 9], though this remains debated [10] due to limited evidence.

Modern imaging modalities, including PET‐CT and ultrasound, particularly of the upper limbs, have markedly improved detection of LV‐GCA, revealing that isolated LV‐GCA without cranial involvement is more common than once thought [8, 10, 11]. Recent PET‐based cohorts report isolated LV‐GCA in up to 20%–30% of diagnosed GCA cases, including 29% in a 2024 Spanish study of 140 patients using 18F‐FDG PET‐CT [8, 9].

GCA with involvement of the mesenteric arteries is rare and limited to case reports [12, 13], although some consider the entity underdiagnosed given documented cases with no or nonspecific symptoms, detected on systemic imaging [5]. GCA‐associated mesenteric ischemia carries high morbidity and mortality, with complications ranging from infarction and bowel perforation to death [5]. In 2008, Scola et al. [13] identified only 11 reported cases of mesenteric involvement in GCA in the English‐language literature—before widespread PET‐CT use—and added their own case (*n* = 12). All patients suffered from abdominal complaints, ranging from acute abdominal pain to chronic postprandial symptoms. Mortality in this study was 50%, with only 3 of the 12 patients having ischemia without infarction.

The patient's diagnosis of LV‐GCA with mesenteric involvement was supported by PET findings, constitutional symptoms, and a prompt biochemical and clinical response to immunosuppression. However, the absence of traditional cranial symptoms, coupled with a longstanding history of functional gastrointestinal disorder, contributed to a significant 6‐month diagnostic delay. This delay highlights the difficulty of distinguishing between chronic functional gut symptoms and the development of subacute mesenteric ischemia. Differentiating between functional symptoms and mesenteric vasculitis requires a high index of suspicion for red flags that signal a transition from a stable functional baseline to a systemic inflammatory process. While functional disorders often present with vague abdominal discomfort, clinicians must recognize that the emergence of severe postprandial pain (abdominal angina), especially when associated with food fear, is suggestive of vascular compromise [5, 14]. In this case, the shift to ischemic‐type pain, when accompanied by systemic constitutional signs such as significant weight loss and night sweats, should have triggered an immediate investigation for an underlying systemic vasculitis.

Standard management involves high‐dose corticosteroids with a prolonged taper [2], while the IL‐6 inhibitor tocilizumab can reduce cumulative steroid exposure and relapse rates [15]. We note the small but significant risk of bowel perforation with tocilizumab, especially when combined with high‐dose prednisolone treatment [16]; however, minimal data exist on the risk of this entity in GCA patients with mesenteric involvement. Multispecialty input in this case was sought from general medicine, rheumatology, gastroenterology, and radiology. In the absence of endoscopic biopsy confirmation or ischemic complications such as perforation, the presentation was felt to be consistent with large‐vessel mesenteric vasculitis without bowel ischemia. At 2 months' follow‐up, the patient remains stable with no recurrence or further intestinal complications.

## 6 Conclusion

Diagnosis of LV‐GCA requires high clinical suspicion given its nonspecific presentation in patients and potential for devastating complications. PET‐CT can be particularly helpful in supporting the diagnosis, especially in cases of undifferentiated high inflammatory states. Although mesenteric involvement in GCA may manifest acutely with life‐threatening surgical complications, clinicians should also recognize subacute postprandial abdominal pain accompanied by constitutional symptoms as a potential presentation.

## Author Contributions

**Benjamin Rao:** conceptualization. **Ronan O'Connor:** conceptualization.

## Funding

The authors have nothing to report.

## Consent

The authors confirm that written patient consent has been signed and collected in accordance with the journal's patient consent policy.

## Data Availability

The data that support the findings of this study provide potentially identifiable information about the patient, and hence will not be made available.
